# Effects of Carvacrol on Aortic Damage in a Streptozotocin-Induced Type 1 Diabetic Rat Model

**DOI:** 10.3390/biom16030431

**Published:** 2026-03-13

**Authors:** Seda Cetinkaya Karabekir, Burcu Gultekin, Hasan Basri Savas, Gokhan Cuce, Serpil Kalkan

**Affiliations:** 1 Department of Histology and Embryology, Faculty of Medicine, Izmir Bakırcay University, Izmir 35665, Turkey; 2 Department of Histology and Embryology, Faculty of Medicine, Necmettin Erbakan University, Meram, Konya 42090, Turkey; 3 Department of Medical Biochemistry, Faculty of Medicine, Mardin Artuklu University, Mardin 47000, Turkey

**Keywords:** diabetes mellitus, oxidative stress, apoptosis, vascular complications, carvacrol, ischemia-modified albumin, STZ-induced diabetes

## Abstract

Diabetes mellitus (DM) is associated with vascular complications that increase morbidity and mortality. Natural antioxidants play a vital role in reducing diabetes-related damage. This study investigated the protective effects of the phenolic monoterpene carvacrol (CAR) against diabetic complications. Thirty-two male Wistar Albino rats (4 months, 250–300 g) were divided into four groups: control, DM, DM + DMSO, and DM + CAR. Type 1 diabetes was induced via intraperitoneal injection of 50 mg/kg streptozotocin (STZ). The DM + CAR group received 20 mg/kg CAR daily for four weeks. Body weight and blood glucose levels were regularly monitored. At the end of the study, aortic tissues were examined using hematoxylin–eosin (H&E), Verhoeff–Van Gieson, and immunohistochemical staining, while cardiac tissues were analyzed with H&E and Masson’s trichrome. Serum levels of ischemia-modified albumin (IMA), cholesterol (CHOL), triglycerides (TG), and high-density lipoprotein (HDL) were measured. In the DM group, IMA and CHOL levels were increased (*p* = 0.0208 and *p* = 0.0207, respectively), apoptosis was elevated (caspase-3 expression, *p* = 0.0001), and marked tissue damage was observed. In contrast, in the DM + CAR group, IMA levels (*p* = 0.0228) and caspase-3 expression (*p* = 0.0457) were reduced, and notable improvements were detected in vascular and cardiac tissues. These results suggest that CAR protects against diabetic complications by modulating oxidative stress, inhibiting apoptosis, and preventing tissue injury.

## 1. Introduction

Type 1 diabetes mellitus (T1DM) is a chronic autoimmune disorder that typically begins in childhood or adolescence and accounts for approximately 10% of all diabetes cases [1]. It is characterized by the autoimmune destruction of pancreatic β-cells, resulting in absolute insulin deficiency and persistent hyperglycemia [2]. Sustained hyperglycemia significantly contributes to systemic complications, especially within the cardiovascular system, by promoting endothelial dysfunction, inflammation, and vascular remodeling [3,4].

Therefore, cardiovascular diseases are among the leading causes of morbidity and mortality in individuals with T1DM, even in the absence of traditional risk factors [5]. One of the most severe complications is diabetic cardiomyopathy (DCM), which is marked by myocardial fibrosis, ventricular hypertrophy, and impaired systolic and/or diastolic function [6]. In this context, oxidative stress plays a central role by aggravating myocardial and vascular damage. Furthermore, ischemia-modified albumin (IMA) is a clinically relevant biomarker for detecting oxidative stress-related diseases such as coronary artery disease, diabetes mellitus, hypercholesterolemia, and metabolic syndrome [7].

Experimental animal models are widely used to investigate the mechanisms underlying T1DM-related complications and develop novel therapeutic approaches. In this context, streptozotocin (STZ) is frequently employed due to its selective toxicity to pancreatic β-cells and its ability to induce clinical and metabolic characteristics resembling those of human T1DM [8].

In current therapeutic strategies, the primary goals in diabetes management are to achieve glycemic control, reduce oxidative stress, and correct abnormalities in lipid metabolism. Therefore, the investigation of natural compounds with potential protective effects remains an important research area [9].

In this context, the therapeutic potential of natural compounds has attracted increasing attention. Aromatic herbs have been widely used in traditional medicine for centuries due to their beneficial and protective properties [10]. One of the major constituents of essential oils derived from these plants is carvacrol (CAR) [2-methyl-5-(1-methylethyl) phenol], a monoterpenoid naturally found in various plants, such as oregano (*Origanum vulgare*), pepper, and wild bergamot. The U.S. Food and Drug Administration (FDA) classifies CAR as safe for use in food products. It has gained attention for its wide range of biological effects, including antibacterial, antifungal, antiviral, immunomodulatory, and antioxidant activities [11]. Owing to its diverse bioactivity, CAR has demonstrated therapeutic potential in various disease models. In particular, its antihypertensive, antilipidemic, and cardioprotective effects offer promising prospects for clinical applications [12]. Dantas et al. (2015) reported that CAR exerts vasorelaxant effects by inhibiting voltage-dependent calcium channels, thereby reducing blood pressure [13]. Moreover, extracts of *Thymbra spicata* var., which contain CAR as a major component, have shown significant anti-hypercholesterolemic, antioxidant, and anti-steatohepatitic effects in high-fat diet-induced obese mice [14]. These findings suggest that CAR may exert protective effects on metabolic and cardiovascular functions.

This study aims to investigate structural and functional alterations in aortic and cardiac tissues induced by Type 1 diabetes and to evaluate the potential protective effects of CAR against these changes. Accordingly, aortic tunica intima thickness will be determined by morphometric analysis, and the cardiovascular impact of diabetes will be comprehensively examined through histopathological evaluations, oxidative-stress markers, apoptotic activity, and cardiac fibrosis analyses. Moreover, since hormonal fluctuations associated with the estrous cycle in female rats can introduce variability in experimental outcomes, and the use of male rats has become the standard approach in STZ-induced diabetes models [9,15], only male Wistar Albino rats were selected to ensure homogeneous results.

## 2. Materials and Methods

### 2.1. Animals and Experimental Groups

A total of 32 male Wistar albino rats, aged four months and weighing 250–300 g, were used in this study. The animals had free access to tap water and standard laboratory chow. The subjects were kept under regulated settings, with a temperature of 24 ± 1 °C, humidity of 45 ± 5%, and a 12-h light/dark cycle.

The rats were randomly assigned to four groups, each consisting of eight animals.

Control Group: No intervention was applied.

DM Group: Type 1 diabetes was induced via streptozotocin (STZ) administration.

DM + DMSO Group: Type 1 diabetes was induced via STZ administration, followed by daily intraperitoneal administration of 0.1% dimethyl sulfoxide (DMSO) (CAS Number: 67-68-5, Sigma-Aldrich, St. Louis, MO, USA) for four weeks.

DM + CAR Group: Type 1 diabetes was induced via STZ administration, followed by daily intraperitoneal administration of 20 mg/kg CAR (Purity: 98%, Sigma-Aldrich: Lot; SHBL6147, St. Louis, MO, USA) dissolved in 0.1% DMSO prepared in physiological saline (0.9% sodium chloride) for four weeks. CAR was prepared before each administration [16,17,18].

The CAR dose (20 mg/kg/day) was selected based on prior studies in STZ-induced diabetes models in which 20 mg/kg/day demonstrated consistent protective effects compared with lower dosing (e.g., 10 mg/kg/day) [16,17,18].

Experimental diabetes was induced via a single intraperitoneal injection of STZ (Sigma-Aldrich: S0130-1G, St. Louis, MO, USA) at a dosage of 50 mg/kg, dissolved in 0.01 M sodium citrate buffer (pH 4.5) [19]. Blood glucose levels were assessed in all groups using a glucometer, with a drop of blood obtained from the tail vein. Rats exhibiting blood glucose levels of 270 mg/dL or above were classified as diabetic. Blood glucose levels were assessed weekly during the experiment [15,20]. In all animals administered with STZ (DM, DM + DMSO, and DM + CAR groups), blood glucose levels exceeding 270 mg/dL were detected within 72 h, confirming the development of diabetes. All 8 rats in each of the three diabetic groups met this criterion.

Before the commencement of the experimental procedures and upon the conclusion of the study, the rats’ body weights were documented. At the end of the four-week trial period, blood samples were collected under anesthesia (50 mg/kg ketamine HCl + 10 mg/kg xylazine HCl), followed by cervical dislocation.

### 2.2. Biochemical Analysis

Upon conclusion of the experiment, blood samples were collected into gel-containing serum separator tubes to facilitate efficient separation of serum from cellular components after centrifugation and centrifuged at 1500× *g* for 10 min to isolate the supernatant serum fraction. The serum samples were subsequently aliquoted into Eppendorf tubes, labeled, and preserved at −80 °C until analysis. Prior to biochemical analysis, all serum samples were concurrently thawed and equilibrated at room temperature. The samples were subsequently vortexed to achieve homogeneity and were prepared for biochemical evaluation.

Serum ischemia-modified albumin (IMA) levels were assessed using the cobalt-binding assay method. For the IMA assessment, 95 µL of serum was mixed with 5 µL of cobalt chloride and incubated for 5 min. The concentration of cobalt chloride was modified to 0.58 mmol/L during incubation. Under ischemic conditions, a minimal amount of cobalt is associated with albumin. To ascertain the unbound cobalt, 25 μL of dithiothreitol (final concentration of 1.67 mmol/L) was introduced post-incubation. The absorbance of this mixture was measured spectrophotometrically at 500 nm. IMA levels were expressed as absorbance units (ABSU) [21,22].

Cholesterol, triglyceride, and HDL levels were measured spectrophotometrically using a biochemical auto-analyzer (AU5800; Beckman Coulter, Inc., Brea, CA, USA) with commercial assay kits according to the manufacturer’s instructions [23].

### 2.3. Hematoxylin-Eosin Staining

After blood sample collection, the abdominal cavities of sacrificed rats were dissected, and the aorta and heart tissues were fixed in 10% formaldehyde for histological analysis. Subsequent to tissue preparation, aortic and cardiac tissues were fixed in paraffin blocks, and 4 µm thick sections were produced using a microtome for microscopic study.

Histopathological and immunohistochemical analyses of the aorta were performed on cross-sections of the thoracic aorta, with evaluation of the tunica intima and tunica media. Cardiac analyses were conducted on left ventricular myocardial sections, focusing on structural alterations and apoptotic activity.

To remove paraffin, the sections were immersed in xylene for three successive 20-min treatments and then passed through a decreasing ethanol gradient (from absolute ethanol down to 50%). They were next stained with hematoxylin–eosin (H&E). After staining, the slides underwent dehydration in an ascending ethanol series (from 50% to absolute ethanol in 10-min increments) before being mounted under coverslips with Entellan. Histopathological analysis was conducted using a Zeiss (Carl Zeiss Microscopy GmbH, Jena, Germany) Lab.A1 light microscope equipped with a Zeiss Axicam ERc 5s camera, and evaluations were performed through a double-blind histopathological examination. Histopathological scores were evaluated using a semi-quantitative system encompassing congestion, connective tissue edema, inflammatory cell infiltration, and vacuolization; for each animal, six randomly selected fields were analyzed and scored as 0 (absent), 1 (mild), 2 (moderate), and 3 (severe).

All histopathological and immunohistochemical evaluations were performed with group allocation concealed by random coding of the specimens. The coding was carried out by a researcher who was not involved in the evaluation process. Histopathological scoring and immunohistochemical assessments were conducted independently by two blinded observers according to the same predefined criteria. Measurements of tunica media thickness in Verhoeff–Van Gieson-stained sections were performed by a single experienced blinded observer to ensure standardization of the measurement protocol. For each animal, six randomly selected fields were evaluated for each staining, and animal-based mean values were used for statistical analysis.

### 2.4. Masson’s Trichrome Staining

Sections measuring 4 μm in thickness were extracted from cardiac blocks of the experimental groups and underwent deparaffinization using xylene (2 × 5 min), followed by a sequential dilution in alcohol (100%, 90%, 80%, 70%, and 50%). The sections were subsequently stained utilizing the BesLab Masson Trichrome Staining Kit (Lot: 072022.036, Ankara, Türkiye). Following a series of rinses with alcohol and xylene, the portions were affixed using Entellan^®^ (Merck KGaA, Darmstadt, Germany).

### 2.5. Verhoeff–Van Gieson Staining

In total, 4 μm-thick sections of aortic tissue were taken and deparaffinized with xylene (3 × 20 min). Subsequently, the sections were stained with the LST GBL Verhoeff–Van Gieson Staining Kit (Ref. No. 5036, GBL – Gul Biology Laboratory Industry and Trade Inc., Istanbul, Turkey) rinsed with an alcohol series and xylene, and mounted with Entellan^®^.

**Figure 1:**
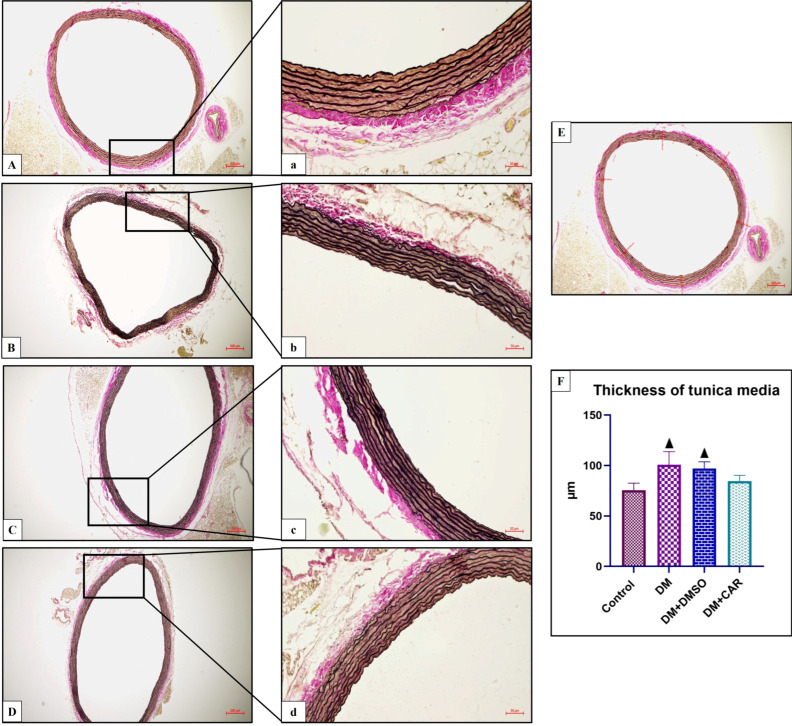
Evaluation of tunica media thickness in the sections of aortic tissues stained with Verhoeff–Van Gieson from all groups. (**A**,**a**); Control (**B**,**b**); DM (**C**,**c**); DM + DMSO (**D**,**d**); DM + CAR. (Verhoeff–Van Gieson (**A**–**D**): ×50; (**a**–**d**): ×200). (**E**) Representative image illustrating the measurement of tunica media thickness. Measurements were performed using Zen Blue 3.4 software, with six randomly selected points along the aorta, and the tunica media thickness was calculated by averaging the measurements obtained from each point. (**F**) ▲ Indicates statistical significance compared to the Control and DM + CAR groups (*p* < 0.05). (Each experimental group consisted of 8 animals). Statistical analysis was performed using one-way ANOVA. Values are presented as mean ± standard deviation. A *p*-value of <0.05 was considered statistically significant.

The tunica media thickness was measured using a light microscope with a 40× objective under Zen Blue 3.4 software. Six random points along the aorta were selected, and the tunica media thickness was calculated as the average of measurements from each point (Figure 1E).

### 2.6. Immunohistochemistry (IHC) Analysis

Aortic tissue sections, 4 µm in thickness, were positioned on lysine-coated slides. The slides were treated in xylene for 30 min, and thereafter underwent antigen retrieval using heat-mediated antigen retrieval in citrate buffer (pH 6.0) for 20 min. A 3% hydrogen peroxide (H_2_O_2_) solution was utilized to inhibit endogenous peroxidase activity. Super Block (ScyTek Laboratories, Logan, UT, USA) was employed for 10 min to inhibit non-specific antigen binding. The sections were treated overnight with caspase 3 (ab184787, Abcam, Cambridge, UK) antibody, subsequently followed by a 20-min incubation with a secondary antibody at room temperature. Between each incubation step, the sections were washed three times with phosphate-buffered saline (PBS) for 5 min. Streptavidin-peroxidase was administered for 20 min. The staining method was executed utilizing 3,3′ diaminobenzidine (DAB) chromogen. The sections were counterstained with Mayer’s hematoxylin once the immunological response became obvious under the microscope. The sections were evaluated using a Zeiss Lab.A1 light microscope, and photos were captured using the Zeiss Axicam ERc 5s camera imaging system.

The immunohistochemical staining results were evaluated according to Cuce et al. (2016) [15] criteria for each primary antibody: 0—no staining, 1—weak staining, 2—moderate staining, 3—strong staining.

**Figure 2:**
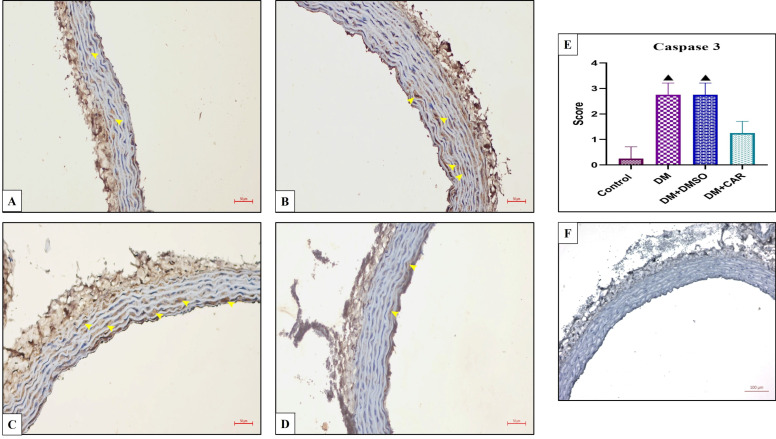
Immunohistochemical staining images of caspase-3 expression in the aortic tissues of all groups. (**A**) Control; (**B**) DM; (**C**) DM + DMSO; (**D**) DM + CAR. Caspase-3–positive staining is indicated by yellow arrows. (DAB-hematoxylin; ×200). (**E**) ▲ Indicates statistical significance compared with the Control and DM + CAR groups (*p* < 0.05). Each experimental group consisted of 8 animals. Statistical analysis was performed using the Kruskal–Wallis test. A *p*-value of <0.05 was considered statistically significant. (**F**) Negative control for immunohistochemical staining.

For the negative control, the primary antibody was omitted, and the sections were incubated with phosphate-buffered saline under the same conditions (Figure 2F).

### 2.7. Statistical Analysis

Statistical processing was performed with GraphPad Prism version 8.

Histological changes and biochemical parameters are reported as the mean ± SD.

Normality of the datasets was confirmed across all groups by Shapiro–Wilk and one-sample Kolmogorov–Smirnov tests. Group comparisons were made using one-way ANOVA. Pairwise differences were then examined with Tukey’s HSD post hoc test. Statistical significance was set at *p* < 0.05. Non-normally distributed immunohistochemical variables were analyzed via Kruskal–Wallis followed by Dunn’s post hoc test.

## 3. Results

### 3.1. Association of CAR Treatment with Aortic Tunica Media Thickness and Elastin Structure

Verhoeff–Van Gieson staining was employed to evaluate the structure of elastin fibers and associated histopathological changes. Histological analysis revealed irregularities in elastin fibers in the DM and DM + DMSO groups. Quantitative analysis of tunica media thickness demonstrated a statistically significant increase in both the DM (*p* < 0.0001 vs. control; *p* = 0.0043 vs. DM + CAR) and DM + DMSO (*p* < 0.0002 vs. control; *p* = 0.0384 vs. DM + CAR) groups compared with the control and DM + CAR groups (Figure 1F). Although an increase in tunica media thickness was observed in the DM + CAR group, this difference was not statistically significant compared to the control group (Figure 1F).

### 3.2. Effects of CAR on Apoptotic Markers in Aortic Tissue of Diabetic Rats

Immunohistochemical analysis of caspase-3 expression revealed a significant increase in both the DM and DM + DMSO groups compared with the control (*p* = 0.0001 for both comparisons) and DM + CAR groups (*p* = 0.0457 for both comparisons). In the DM + CAR group, caspase-3 expression was higher than in controls, but this increase did not reach statistical significance (*p* = 0.7176) (Figure 2E).

### 3.3. Effects of Diabetes and CAR on Aortic Wall and Heart Histopathology

**Figure 3:**
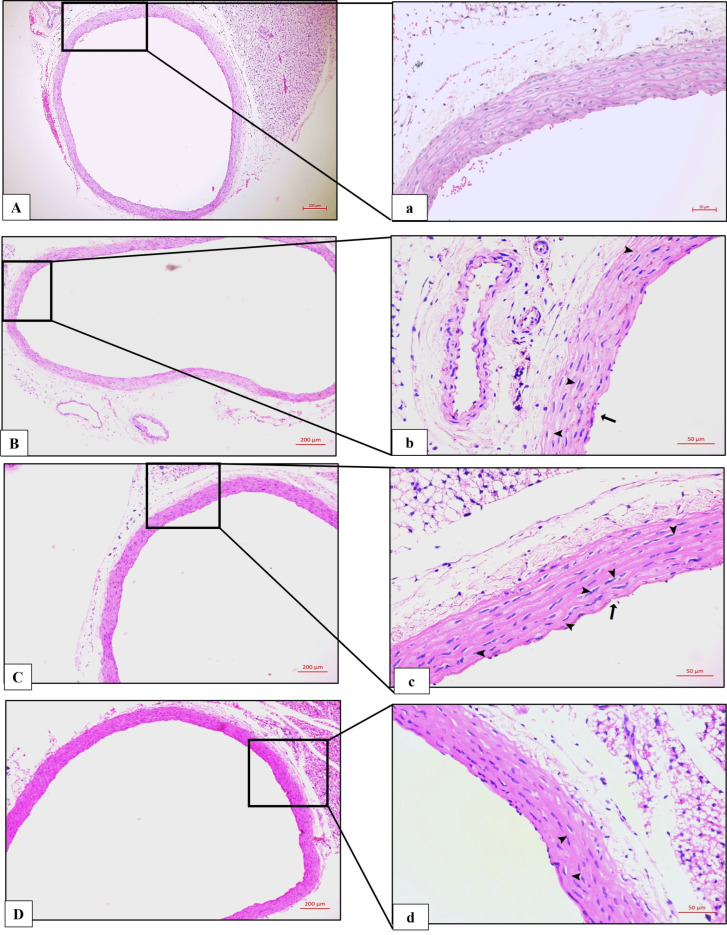
Histopathological evaluation of H&E-stained aortic tissue sections from all groups. (**A**,**a**) Control; (**B**,**b**) DM; (**C**,**c**) DM + DMSO; (**D**,**d**) DM + CAR. Damage in the endothelial layer, denudation of the intimal surface (black arrow), and vacuolization (arrowhead) are shown. (**A**–**D**) H&E ×50; (**a**–**d**) H&E ×200. (Each experimental group consisted of 8 animals).

In the histological evaluation with H&E staining, the control group aortic sections exhibited normal structure, including the tunica intima, tunica media containing elastic fibers, and the tunica adventitia layers (Figure 3A,a). Compared with the control group, both the DM and DM + DMSO groups showed significant sclerotic changes in the aortic wall, atrophy of elastic fibers, endothelial cell loss in the tunica intima, structural irregularities in the tunica media and tunica adventitia, and vacuolization (Figure 3B,b,C,c). In the DM + CAR group, it was found that these histopathological changes were significantly reduced compared to the DM and DM + DMSO groups (Figure 3D,d).

**Figure 4:**
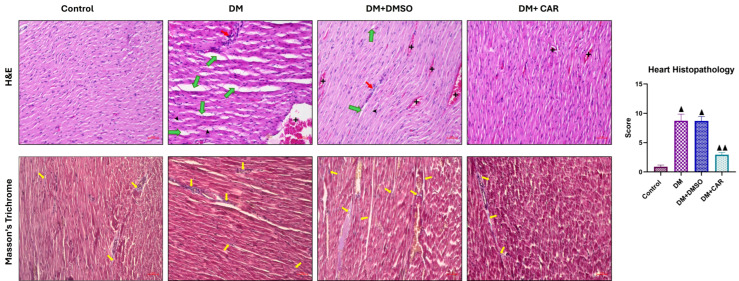
Histopathological evaluation of H&E- and Masson’s Trichrome-stained sections of heart tissues from all groups. Edema in the connective tissue (green arrow), congestion (+), inflammatory cell infiltration (red arrow), and vacuolization (head of the arrow) are indicated. (H&E and Masson’s Trichrome ×200). ▲ Indicates statistical significance compared to the Control group; ▲▲ Indicates statistical significance compared to the Control, DM, and DM + DMSO groups (*p* < 0.05). (Each experimental group consisted of 8 animals). Statistical analysis was performed using one-way ANOVA. Values are presented as mean ± standard deviation. A *p*-value of <0.05 was considered statistically significant.

The typical architecture of myofibrils, characterized by striation, a branching appearance, and continuity with neighboring myofibrils, was preserved in the control group cardiac sections (Figure 4). In the DM and DM + DMSO groups, congestion, edema in the connective tissue, infiltration of inflammatory cells, and vacuolization were noted. The results indicated a statistically significant increase relative to the control group (*p* < 0.0001) (Figure 4) within the DM + CAR cohort. A significant rise in histopathological changes was noted relative to the control group (*p* < 0.0001), and these changes exhibited a statistically significant reduction compared to the DM and DM + DMSO groups (Figure 4).

### 3.4. Assessment of Cardiac Collagen by Masson’s Trichrome Staining

Histological evaluation using Masson’s Trichrome staining revealed that collagen fiber density in the heart tissues of the control group rats exhibited a normal distribution (Figure 4). In the DM and DM + DMSO groups, a marked increase in collagen fiber density and distribution was observed compared to the control and DM + CAR groups (Figure 4). Conversely, the DM + CAR group showed a decrease in collagen fiber density relative to the DM and DM + DMSO groups (Figure 4).

### 3.5. Effects of CAR on Oxidative Stress and Lipid Parameters in Diabetic Rats

**Figure 5:**
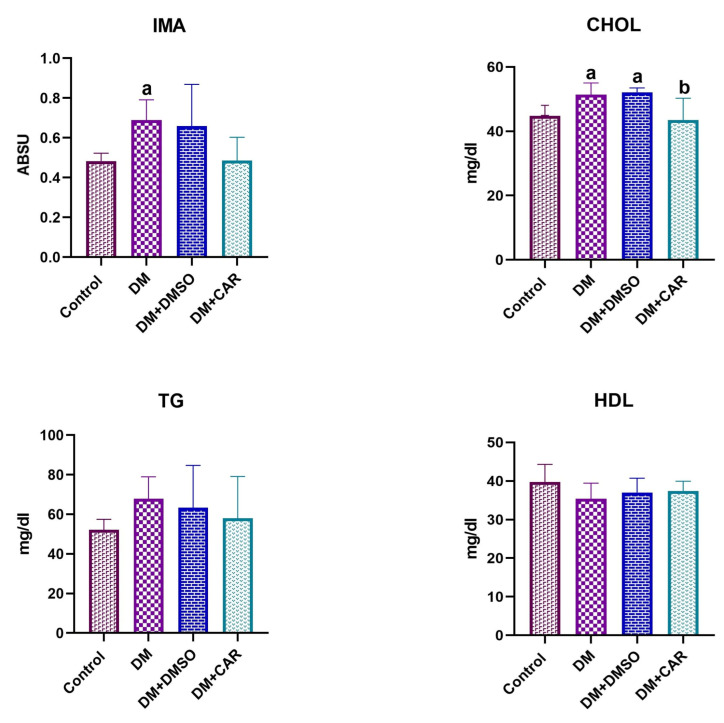
Serum IMA, CHOL, TG and HDL levels in each experimental group (mean ± SD; n = 8). One-way ANOVA was used for statistical comparison, with *p* < 0.05 deemed significant. a—indicates statistical significance when compared to the control and DM + CAR groups. b—indicates statistical significance when compared to the DM and DM + DMSO groups (*p* < 0.05). (Each experimental group consisted of 8 animals).

Biochemical parameters (IMA, CHOL, TG, and HDL) were analyzed across all groups. TG and HDL concentrations remained comparable across the experimental cohorts. By contrast, IMA levels were significantly elevated in the DM group relative to the control (*p* = 0.0208) and DM + CAR (*p* = 0.0228) groups. Total cholesterol (CHOL) rose markedly in the DM group versus the control (*p* = 0.0207) and DM + CAR (*p* = 0.0048) groups. Additionally, the DM + DMSO cohort exhibited elevated CHOL compared to the control (*p* = 0.0087) and DM + CAR (*p* = 0.0019) groups. Conversely, CHOL levels in the DM + CAR cohort were restored to control values, showing no significant difference (*p* = 0.9349). This normalization of CHOL by CAR indicates its potential to mitigate diabetes-induced hypercholesterolemia (Figure 5).

## 4. Discussion

This study demonstrated that streptozotocin-induced diabetes in rats adversely affected the cardiovascular system through multiple pathological mechanisms, including increased oxidative stress, enhanced apoptosis, disturbances in lipid metabolism, and marked histopathological alterations. Notably, CAR treatment significantly alleviated these alterations, underscoring its potential cardioprotective and vasoprotective properties.

Diabetes mellitus (DM) is a chronic disease that promotes atherosclerosis and vascular dysfunction, leading to both macrovascular and microvascular complications [24,25]. Oxidative stress induced by prolonged hyperglycemia is recognized as a key underlying mechanism. Hyperglycemia facilitates the accumulation of advanced glycation end-products (AGEs), promotes the formation of atherogenic intermediate-density lipoproteins (IDL), and impairs both the anti-inflammatory properties and reverse cholesterol transport function of high-density lipoproteins (HDL) [26]. In addition, persistent hyperglycemia and insulin deficiency exacerbate systemic inflammation, endothelial dysfunction, and atherosclerosis [27]. Elevated levels of reactive oxygen species (ROS) due to hyperglycemia, along with PKC activation, increased superoxide dismutase (SOD) production, and decreased nitric oxide (NO) availability in vascular smooth muscle cells, collectively impair endothelial function and contribute to cardiomyocyte apoptosis and myocardial fibrosis [25,28,29,30]. In vitro studies have confirmed that high-glucose exposure induces oxidative damage in cardiomyocytes, endothelial cells, and neurons, and STZ-induced diabetic animal models have demonstrated increased oxidative stress and apoptosis [31,32,33]. Consistent with these reports, our results showed elevated oxidative stress in diabetic rats, evidenced by significantly higher serum ischemia-modified albumin (IMA) levels in the DM group. By contrast, IMA levels in the DM + CAR group were comparable to those in controls, indicating that CAR effectively mitigates oxidative stress and associated cellular injury.

Regarding lipid metabolism, our findings partially differ from those reported by Ramadan et al. (2021) [34]. In that study, increased triglyceride levels were reported in STZ-induced diabetic rats. In our study, however, TG and HDL levels did not change across the experimental groups, whereas total cholesterol (CHOL) levels were markedly elevated in the DM group. This finding may be associated with increased intestinal cholesterol absorption related to insulin deficiency [35]. The significant CHOL reduction in the DM + CAR group suggests that CAR exerts an antihyperlipidemic effect, potentially lowering cardiovascular risk.

Our apoptosis analysis further supports the role of programmed cell death in diabetic complications. Elevated caspase-3 expression in the DM group mirrors the findings of Cuce et al. (2016) [15], who reported increased pro-apoptotic markers in vascular smooth muscle cells of diabetic rats. CAR treatment markedly reduced caspase-3 expression, indicating its anti-apoptotic potential.

Histopathological analyses of vascular tissue revealed structural abnormalities similar to those reported by Budin et al. (2009) [36] and Elbe et al. (2014) [37], including tunica media thickening, disrupted elastic fibers, endothelial loss, and elastic lamella degradation. These changes were substantially attenuated in the DM + CAR group, suggesting preservation of vascular integrity.

Cardiac tissue examination further confirmed the protective effects of CAR. In line with previous studies [38,39], DM and DM + DMSO groups exhibited cardiomyocyte degeneration, interstitial hemorrhage, collagen accumulation, and inflammatory infiltration. CAR treatment significantly reduced fibrosis and edema while preserving myocardial architecture.

This study has certain limitations. First, only male rats were used, limiting the extrapolation of results to both sexes. Future studies including female subjects may provide a more comprehensive evaluation of treatment effects. Second, only a single dose of CAR was tested; assessing multiple dose levels would help determine an optimal therapeutic range. Third, the relatively short treatment period (4 weeks) restricts conclusions regarding long-term effects. Longer follow-up studies are needed to assess the durability of CAR’s benefits.

## 5. Future Perspectives

While interpreting these findings, it should be considered that animal and human doses cannot be directly compared on a mg/kg basis; therefore, no clinical dose recommendation is made. According to body surface area-based scaling recommended by the Food and Drug Administration (FDA) (2005), a CAR dose of 20 mg/kg administered in rats corresponds to approximately 3.24 mg/kg in humans (≈195–260 mg/day for a 60–80 kg individual) [40]. Although this value is higher than the 1–2 mg/kg/day doses reported to be tolerated in short-term oral administration in humans [41], the human equivalent dose (HED) is used solely to provide an approximate indication of interspecies exposure. Therefore, translation to clinical application will require dedicated pharmacokinetic and safety studies.

## 6. Conclusions

This study demonstrated that CAR exerts significant protective effects on the aortic and cardiac tissues of streptozotocin-induced type 1 diabetic rats. CAR treatment reduced oxidative stress, suppressed apoptosis, normalized cholesterol levels, and mitigated histopathological alterations. These findings suggest that CAR may represent a potential therapeutic approach for preventing diabetes-related cardiovascular complications. However, given the limitations of using a single dose, short duration, and only male animals, further long-term studies with different doses and both sexes are recommended to better evaluate its efficacy, safety, and translational potential in clinical practice.

## Data Availability

All data generated or analyzed during this study are included in this article. Further inquiries can be directed to the corresponding author.
